# Basic COVID-19 knowledge according to education level and country of residence: Analysis of twelve countries in Latin America

**DOI:** 10.3389/fmed.2022.978795

**Published:** 2022-09-29

**Authors:** Christian R. Mejia, Telmo Raul Aveiro-Robalo, Luciana Daniela Garlisi Torales, Maria Fernanda Fernández, Francisco E. Bonilla-Rodríguez, Enrique Estigarribia, Johanna Magali Coronel-Ocampos, Cecilia J. Caballero-Arzamendia, Renato R. Torres, Aram Conde-Escobar, Yuliana Canaviri-Murillo, Diana Castro-Pacoricona, Victor Serna-Alarcón, Dennis Arias-Chávez

**Affiliations:** ^1^Translational Medicine Research Centre. Universidad Norbert Wiener, Lima, Peru; ^2^Universidad del Pacífico, Asunción, Paraguay; ^3^Universidad Privada de Tacna, Centro de Investigación de Estudiantes de Medicina (CIESMED), Tacna, Peru; ^4^Asociación Médica de Investigación y Servicios en Salud, Lima, Peru; ^5^José Matías Delgado University, La Libertad, El Salvador; ^6^National University of Caaguazú, Coronel Oviedo, Paraguay; ^7^Universidad Católica Nuestra Señora de la Asunción, Guairá, Villarrica, Paraguay; ^8^Antenor Orrego Private University, Trujillo, Peru; ^9^Hospital José Cayetano Heredia, Piura, Peru; ^10^Universidad Continental, Arequipa, Peru

**Keywords:** coronavirus, knowledge, pandemic, COVID-19, Latin America

## Abstract

**Introduction:**

Knowing a disease is crucial for being able to fight it, especially in a region in which COVID-19 caused so many deaths, such as Latin America.

**Objective:**

To determine the association between basic knowledge of COVID-19 and education level according to country of residence in Latin America.

**Methodology:**

This is an analytical cross-sectional study. Basic level of knowledge was measured through nine close-ended questions (scale validated in Peru). The score obtained was analyzed through performing a crosstab vs. gender, age, education level, and country of residence.

**Results:**

Of a total of 9,222 respondents, almost all of them knew the common symptoms (99%), modes of transmission (93%), and knew how to recognize which was not a specific symptom (93%). Through the multivariate model, we found that there was no association with gender (*p* = 0.716) or age (*p* = 0.059), in comparison with those who had primary or a lower education level. All the other higher education levels had statistically significant scores (all p-values *p* < 0.001). When comparing knowledge according to countries, and using Peru as reference for comparison, Chile, Paraguay, Mexico, Bolivia, Panama, Ecuador, Costa Rica, and Colombia had a better level of knowledge (all *p*-values < 0.001); however, only El Salvador had a lower level (*p* < 0.001).

**Discussion:**

There was lack of knowledge of some topics, difference according to academic degree and country. As Peru was one of countries that obtained the lowest level of knowledge, it could have influenced the fact that it was the most affected country in the world.

## Introduction

COVID-19 is a disease caused by SARS-CoV-2, discovered in the city of Wuhan, China, at the end of 2019 (1). This new disease was declared a pandemic by the World Health Organization (WHO) at the beginning of 2020, and it became one of the most important in recent times (2). Nobody can deny the great impact that COVID-19 had worldwide. It caused several repercussions which affected quality of life, health of populations, the economy of families and countries, among many others (2, 3).

The information and knowledge that we currently have about COVID-19 has arisen based on the research carried out, and different topics, such as pathophysiology, clinical manifestations, and evolution have become better known (4, 5). However, despite the fact that this disease has been present in our environment for more than 2 years, it is necessary to see retrospectively what could have influenced some countries to be more affected than others. Moreover, it is important to know that mortality can also vary according to the stage of the disease, the territory or country, the population group, as well as pre-existing comorbidities (6, 7).

In other pathologies there was minimal knowledge to fight them, such as MERS and SARS (8). Therefore, effectively dealing with this disease would have meant, at least, having general knowledge, such as the modes of transmission, the main symptoms, the population at risk, among other information, which can be measured with rapid and effective tests (9, 10). Therefore, it is necessary to know how Latin America faced the disease according to its level of knowledge, as it was one of the most affected regions in the world (11). We can add also that there was a large amount of low quality information that was shared in the media in this region (12). For this reason, the objective of this research was to determine the association between basic knowledge of COVID-19 and education level according to country of residence in Latin America.

## Methods

### Research type and design

During June, July, and August 2020, we undertook an analytical and multicenter study with a cross-sectional observational design. We used a virtual survey, since in those months, the population was experiencing mandatory quarantine in most of the countries; hence, we had to use virtual resources in order to administer the survey.

### Population and sample

We included population living in any of the countries during the pandemic and who could fill in the survey. Those who did not answer completely the questions about knowledge level and secondary variables were excluded (1,274 eliminated surveys). It should be remarked that duplicate surveys, those with incoherent responses, and those with repetitive patterns were detected through a review process by 3 different authors independently and were not taken into account at the time of debugging.

A sample size calculation was performed to find a hypothetical minimum difference of 1.5% (49% vs. 50.5%), for which a minimum of 8,719 respondents was required, with a power of 80%, a confidence level of 95% and for a single sample (due to the analytical cross-sectional design). The final sample had 9,222 respondents; this number was reached through non-random sampling.

### Instruments and procedures

In order to assess the level of knowledge of the 12 Latin American countries, the COVID−19 knowledge scale was used (10). It measures knowledge about basic aspects of coronavirus, such as mortality, vulnerable populations according to mortality, and modes of transmission. This was assessed through nine multiple-choice questions. The scale was validated in Peru during the first months of the pandemic and showed good values of comprehensibility, validity, and reliability.

This survey was composed of other questions that would serve to characterize the population, as well as other adjustment variables for the analytical section. These variables were gender (dichotomous variable with the following possible answers: male or female), age (quantitative variable in years), the highest level of education they had (primary or lower, secondary, bachelor's degree, technical, college/university, and postgraduate), as well as each of the 12 countries where the respondents said they lived (Peru, Chile, Mexico, Paraguay, Colombia, Bolivia, Panama, Ecuador, Costa Rica, El Salvador, Honduras and Guatemala).

All these questions were uploaded to a survey on the Google Forms platform. It could be distributed to all Latin American countries, through each of the authors and the FELSOCEM-ASOMEDISS COVID-19 Latam network, which generated a research group based on their contacts in each of the countries associated with this scientific collaboration network.

### Statistical analysis

For the statistical analysis, first we had to obtain the frequencies and percentages of each one of the test's answers. Then, we calculated the percentage of correct answers by each country; here we could obtain *p*-values (we determined if there was a difference among the percentages of answers by each country, for which the chi-squared test was used). A box-and-whisker plot was also generated to compare the scores obtained in the test according to each level of education evaluated (a *p*-value was also obtained here, with the Kruskal Wallis statistical test).

Finally, the crude and adjusted model was obtained (a model adjusted for sex and age was obtained, and another without these variables, since, although they were not statistically significant, we wanted to see their influence on the other variables). To this end, linear regression was used, taking the score that each respondent obtained as the dependent variable. This score was based on the test of basic level of knowledge of COVID-19. For each of the statistical crosses, a *p*-value < 0.05 was considered statistically significant.

### Ethical aspects

The ethical precepts of the research were respected at all times. After the project was set up, it was submitted to an institutional ethics committee, which evaluated and approved the protocol (resolution of the bioethics committee N°0233-2020-UPAO). It is important to mention that it was approved only by one institution, since the project was generated in April, when almost all the countries' educational institutions were not in operation or everything was paralyzed; therefore, each site agreed to participate with the current ethics committee of the main site of the research.

A consent form was sent with each virtual survey which was signed before filling out the survey. In addition, it was indicated that they were free to participate in the research, to answer the questions they wished, and that their participation was completely anonymous.

## Results

Out of the 9,222 respondents in Latin America, 59.2% (5,455) were women, with a median of 22 years old (interquartile range: 20–30 years old). Regarding education level, 101 (1.1%) had primary or a lower level of education; 1,268 (13.68%), secondary education; 750 (8.1%), a bachelor's degree; 804 (8.7%), technical studies; 5,668 (61.5%), higher education; and 631 (6.8%), postgraduate studies. Almost the total of the respondents knew the common symptoms (98.6%), the modes of transmission (92.9%), and knew how to recognize which was not a specific symptom (92.5%). However, the lowest level of knowledge was about the percentage of mortality of this disease (40.0%), how to manage the symptoms (53.6%), and the treatment of a non-severe presentation of the disease (56.3%) (Table 1).

**Table 1:** Percentage of correct answers by each one of the nine questions of basic knowledge of COVID-19 in 12 countries in Latin America.

**Question**	**Correct *n*(%)**
1. How is the coronavirus transmitted or what is the mechanism of transmission? Answer: Airborne transmission	8,569 (92.9 %)
2. How long is the incubation period or how soon can symptoms of coronavirus manifest? Answer: Up to 14 days.	7,122 (77.2 %)
3. Which are the common symptoms that a person infected with coronavirus could have? Answer: The same symptoms as the flu/a cold's.	9,091 (98.6 %)
4. Which of the following is not one of the most common symptoms of coronavirus infection? Answer: Diarrhea	8,529 (92.5 %)
5. What is the probability of dying (mortality rate) from coronavirus in the general population? Answer: Lower than 5%	3,686 (40.0 %)
6. Who are at a highest risk because of the coronavirus mortality rate? Answer: Older adults	8,113 (88.0 %)
7. What treatment should be given to a person who has initial (non-severe) coronavirus infection? Answer: Treatment should calm respiratory symptoms.	5,192 (56.3 %)
8. What is the diagnostic method used to confirm a coronavirus infection? Answer: Nasal and/or buccal swabbing	7,990 (86.6 %)
9. What would you do if you have symptoms of a cold and suspect you are infected with coronavirus? Answer: I would stay at home until I can recover.	4,939 (53.6 %)

When the percentages of correct answers were broken down by country, it was observed that the Central American countries had the lowest percentages of correct answers. Among them, El Salvador had the lowest percentages in four of the questions, and Costa Rica had the lowest percentages in two of the questions. Other countries with low answers were Bolivia, Mexico and Guatemala (each with a low percentage) (Table 2).

**Table 2:** Percentage of correct answers by each one of the nine questions of basic knowledge of COVID-19 in 12 countries in Latin America.

**Country**	**Percentage of correct answers of the questions**									
	**1**	**2**	**3**	**4**	**5**	**6**	**7**	**8**	**9**	**Mean (SD)**
Peru	91%	72%	98%	91%	34%	83%	48%	84%	58%	6.6 (1.4)
Chile	98%	83%	99%	97%	48%	97%	73%	90%	56%	7.4 (1.2)
Paraguay	95%	81%	99%	96%	53%	92%	81%	96%	55%	7.5 (1.2)
Mexico	96%	85%	99%	93%	26%	96%	55%	85%	59%	6.9 (1.3)
Bolivia	94%	87%	99%	92%	44%	96%	60%	79%	41%	6.9 (1.3)
Panama	96%	90%	100%	96%	67%	97%	69%	98%	36%	7.5 (1.0)
Ecuador	97%	85%	99%	93%	47%	93%	63%	81%	59%	7.2 (1.3)
Costa Rica	94%	77%	97%	93%	54%	81%	68%	94%	27%	6.9 (1.3)
El Salvador	84%	65%	99%	84%	35%	83%	46%	92%	31%	6.2 (1.5)
Honduras	95%	87%	97%	94%	40%	93%	47%	93%	31%	6.8 (1.2)
Colombia	98%	86%	99%	91%	58%	95%	78%	90%	60%	7.6 (1.3)
Guatemala	97%	70%	96%	90%	41%	85%	53%	98%	39%	6.7 (1.5)
*P*-value	<0.001	<0.001	0.001	<0.001	<0.001	<0.001	<0.001	<0.001	<0.001	6.9 (1.4)^*^

P-values were obtained with the chi-squared test. Mean and standard deviation (SD) are of the average score for each country.

* This is the global average.

**Figure 1:**
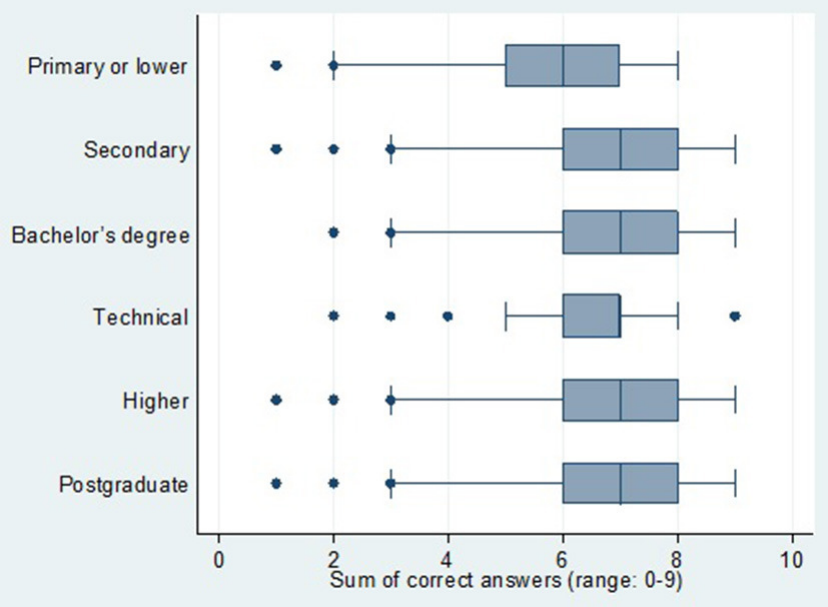


The median of the correct answers was lower among those who had primary or a lower level of education. In the other education levels, we could obtain the same median and similar interquartile ranges (p-value < 0,001 with Kruskal Wallis test) (Figure 1).

When performing multivariate model for the test score, we found that there was no association of gender (*p* = 0.716) or age (*p* = 0.059), in comparison with those who had primary or lower education. All the other education levels had statistically significant scores (all *p*-values were < 0.001. When comparing the knowledge according to country, and using Peru as reference for comparison, Chile, Paraguay, Mexico, Bolivia, Panama, Ecuador, Costa Rica and Colombia had a better level of knowledge (all *p*-values were < 0.001); however, only El Salvador had a lower level *p* < 0.001) (Table 3).

**Table 3:** Bivariate and multivariate models of the basic socio-educational factors associated with the score of basic knowledge of COVID-19 in 12 countries in Latin America.

**Variables**	**Models (knowledge score as a dependent variable)**		
	**Bivariate**	**Multivariate 1**	**Multivariate 2**
**Gender**	−0.04(−0.10/ 0.01) 0.128	Not included in the model	−0.01(−0.06/ 0.04) 0.716
Age (years)	<-0.01(<-0.01/ < 0.01) 0.118	Not included in the model	<-0.01(<-0.01/ < 0.01) 0.059
**Education**			
Primary or lower	Reference category	Reference category	Reference category
Secondary	0.82(0.54/ 1.10) < 0.001	0.88(0.61/ 1.15) < 0.001	0.85(0.58/ 1.12) < 0.001
Bachelor's degree	1.01(0.73/ 1.29) < 0.001	0.90(0.63/ 1.18) < 0.001	0.87(0.59/ 1.15) < 0.001
Technical	0.74(0.46/ 1.02) < 0.001	0.77(0.49/ 1.04) < 0.001	0.75(0.48/ 1.03) < 0.001
Higher	1.16(0.89/ 1.42) < 0.001	1.07(0.81/ 1.33) < 0.001	1.04(0.78/ 1.30) < 0.001
Postgraduate	1.40(1.11/ 1.68) < 0.001	1.32(1.04/ 1.60) < 0.001	1.33(1.05/ 1.60) < 0.001
**Country of residence**			
Peru	Reference category	Reference category	Reference category
Chile	0.81(0.71/ 0.90) < 0.001	0.78(0.69/ 0.88) < 0.001	0.79(0.70/ 0.89) < 0.001
Paraguay	0.89(0.79/ 0.99) < 0.001	0.84(0.73/ 0.94) < 0.001	0.83(0.73/ 0.94) < 0.001
Mexico	0.34(0.23/ 0.46) < 0.001	0.31(0.19/ 0.43) < 0.001	0.30(0.18/ 0.42) < 0.001
Bolivia	0.33(0.21/ 0.46) < 0.001	0.28(0.16/ 0.41) < 0.001	0.28(0.15/ 0.40) < 0.001
Panama	0.88(0.75/ 1.01) < 0.001	0.82(0.68/ 0.95) < 0.001	0.81(0.68/ 0.94) < 0.001
Ecuador	0.56(0.41/ 0.71) < 0.001	0.56(0.40/ 0.71) < 0.001	0.55(0.40/ 0.70) < 0.001
Costa Rica	0.25(0.08/ 0.43) 0.005	0.32(0.14/ 0.49) < 0.001	0.33(0.15/ 0.50) < 0.001
El Salvador	−0.41(−0.58/ −0.24) < 0.001	−0.43(−0.59/ −0.26) < 0.001	−0.42(−0.59/ −0.25) < 0.001
Honduras	0.16(−0.02/ 0.35) 0.086	0.12(−0.07/ 0.30) 0.211	0.11(−0.07/ 0.30) 0.240
Colombia	0.97(0.76/ 1.20) < 0.001	0.92(0.69/ 1.15) < 0.001	0.92(0.69/ 1.15) < 0.001
Guatemala	0.09(−0.16/ 0.35) 0.477	0.06(−0.20/ 0.31) 0.668	0.06(−0.20/ 0.31) 0.649

The models were obtained with linear regression. The score of each respondent obtained with the test for the level of basic knowledge of COVID-19 was taken as a dependent variable. Left of parentheses: Regression coefficient. Inside the parentheses: Confidence interval at 95% (CI 95%). Right of parentheses: p-value.

## Discussion

The good implementation of sanitary measures was fundamental to control the spread of COVID-19. However, success depended largely on the mutual efforts of the population and their governments. In addition, a good level of knowledge of the population (13), as well as a good management of resources to possess the essential tools that help us face the disease was also imperative (14). In this study it was found that, although the surveyed population had good percentages of knowledge about the symptoms and route of transmission of COVID-19, the percentages of knowledge about how to act in case of suspicion of this disease, or of a non-severe presentation, were still not adequate. Thus, people considered that they should go to hospitals or health care centers, even if they had no symptoms or if these were mild. This could have led to the collapse of the health system in many countries, due to an excessive increased demand (15). It also led to self-medication with drugs that did not have sufficient scientific evidence (16), or that, in certain populations, could cause the disease to worsen (17).

On the other hand, in some countries such as El Salvador and Costa Rica, there was a lower number of correct answers, and although these results cannot be extrapolated in a conclusive way, this shows that each country should evaluate the knowledge that their population groups have at the moment of a serious event. This difference between Central American countries and the other Latin American countries may be due to the different information disseminated by the authorities of each country, as well as how they understand it (18–20). Therefore, it is important for governments and authorities to disseminate accurate information during a health crisis, such as the one produced by this pandemic, in print, social and electronic media. This information should be in the local languages of the population, because a better knowledge of the disease will allow people to take appropriate measures and remain calm (21).

In regard to educational level, it was found that those with a higher or postgraduate level of education had a better level of knowledge compared to those with only a primary or a lower level. This result is similar to what was reported by multiple studies, in which a better education level was associated with having correct ideas about COVID-19 (22–24). Therefore, governments and authorities should intensify information dissemination campaigns in the population with primary or lower educational levels, as well as implement measures to reduce the dissemination of false information. Thus, we could prevent this from hindering decision making and influencing negatively, generating excessive fear, or on the contrary, denying the existence of the disease or demanding the non-use of biosecurity measures, which favors the spread of the virus (25). This is especially true in Latin America, where there was an increase in false news as the pandemic progressed (26).

In several countries there was a better knowledge of this new disease, compared to Peru. This reality could have influenced the situation that took place in this country in July and August 2020, when it was considered the most affected country by the pandemic worldwide, according to mortality rates (27). This situation occurred despite the prevention measures adopted by the Peruvian government, such as social distancing and quarantine, and it was reported that these measures were not fully complied with by the population (28). Similarly, this study found that, in Peru, there was a lack of knowledge of COVID-19 mortality rate. This could be explained by the lack of transparency in reporting deaths from this disease, as when the number of deaths from COVID-19 was revealed, it turned out to be almost three times the number reported in the official count (29).

Regarding the low percentage of knowledge of the actions that should be taken in the event of suspected infection, these results could be explained by the deficient information provided by the Peruvian authorities regarding the management of this new disease (30). In addition, we can mention the population's fear of becoming infected, and the misinformation spread by some physicians when encouraging the use of drugs, such as Ivermectin or Warfarin, to prevent COVID-19 (31). All this was reflected in the saturation of health services in the country (32). Therefore, this evidence should be taken into account to improve the management of a health crisis, or any other type of crisis. These results could explain, partially, what was experienced. It is always necessary to inform the population adequately, since ignorance and uncertainty will always be the worst enemy in times of chaos.

The main limitation of the research was selection bias, since, due to the non-random sampling, it cannot be said that what was shown represents the knowledge of each of the countries surveyed or of the whole Latin America. However, this was already foreseen from the conception of the research, since the objective was to find specific associations (it was never to generate a research that could extrapolate the results to the territories mentioned, due to the aforementioned technical difficulties arisen during quarantine). Despite this limitation, we have a very large sample of respondents in a dozen Latin American countries, where the vast majority belong to the most affected country in the world (with the highest mortality per hundred thousand inhabitants in July and August) (27). Hence, it is considered that these results should be taken with the reservation, to show the knowledge that the surveyed populations had in the hardest months of the pandemic.

Based on the results, it is concluded that the best knowledge of the disease was about common symptoms, mode of transmission, and recognition of which was not a specific symptom. There was no association between the knowledge score with gender or age, but there was an association with education level and country of residence.

## Data availability statement

The raw data supporting the conclusions of this article will be made available by the authors, without undue reservation.

## Ethics statement

The studies involving human participants were reviewed and approved by Universidad Privada Antenor Orrego bioethics committee. The patients/participants provided their written informed consent to participate in this study.

## Author contributions

CM, TA-R, and LG contributed to conception and design of the study. LG and TA-R organized the database. CM performed the statistical analysis. TA-R wrote the first draft of the manuscript. MF, FB-R, EE, JC-O, CC-A, RT, AC-E, YC-M, DC-P, VS-A, and DA-C wrote sections of the manuscript. All authors contributed to manuscript revision, read, and approved the submitted version.

## Conflict of interest

The authors declare that the research was conducted in the absence of any commercial or financial relationships that could be construed as a potential conflict of interest.

## Publisher's note

All claims expressed in this article are solely those of the authors and do not necessarily represent those of their affiliated organizations, or those of the publisher, the editors and the reviewers. Any product that may be evaluated in this article, or claim that may be made by its manufacturer, is not guaranteed or endorsed by the publisher.
